# Blood Urea Nitrogen as a Prognostic Marker in Severe Acute Pancreatitis

**DOI:** 10.1155/2022/7785497

**Published:** 2022-03-29

**Authors:** Minhui Dai, Yifei Fan, Pinhua Pan, Yun Tan

**Affiliations:** ^1^Center of Respiratory Medicine, Xiangya Hospital, Central South University, Changsha, 410008 Hunan, China; ^2^Hunan Engineering Research Center for Intelligent Diagnosis and Treatment of Respiratory Disease, Changsha 410008, China; ^3^National Key Clinical Specialty, Branch of National Clinical Research Center for Respiratory Disease, Xiangya Hospital, Central South University, Changsha 410008, China; ^4^National Clinical Research Center for Geriatric Disorders, Xiangya Hospital, Changsha 410008, China; ^5^Critical Care Medicine, Hunan Prevention and Treatment Institute for Occupational Diseases, Changsha, Hunan, China 410007

## Abstract

**Objectives:**

To explore independent risk factors with good and early predictive power for SAP severity and prognosis.

**Methods:**

Patients with SAP were enrolled at Central South University Xiangya Hospital between April 2017 and May 2021 and used as the training cohort. From June 2021 to February 2022, all patients with SAP were defined as external patients for validation. Patients were grouped by survival status at a 30-day posthospital admission and then compared in terms of basic information and laboratory tests to screen the independent risk factors.

**Results:**

A total of 249 patients with SAP were enrolled in the training cohort. The all-cause mortality rate at a 30-day postadmission was 25.8% (51/198). Blood urea nitrogen (BUN) levels were significantly higher in the mortality group (20.45 [interquartile range (IQR), 19.7] mmol/L) than in the survival group (6.685 [IQR, 6.3] mmol/L; *P* < 0.001). After propensity score matching (PSM), the BUN level was still higher in the mortality group than in the survival group (18.415 [IQR, 19.555] mmol/L vs. 10.63 [IQR, 6.03] mmol/L; *P* = 0.005). The area under the curve (AUC) of the receiver operating characteristic curve (ROC) of BUN was 0.820 (95% confidence interval, 0.721–0.870; *P* < 0.001). The optimal BUN level cut-off for predicting a 30-day all-cause mortality was 10.745 mmol/L. Moreover, patients with SAP were grouped according to BUN levels and stratified according to optimal cut-off value. Patients with high BNU levels were associated with significantly higher rates of invasive mechanical ventilation (before PSM: 61.8% vs. 20.6%, *P* < 0.001; after PSM: 71.1% vs. 32%, *P* = 0.048) and a 30-day all-cause mortality (before PSM: 44.9% vs. 6.9%, *P* < 0.001; after PSM: 60% vs. 34.5%, *P* = 0.032) than those with low BNU levels before or after PSM. The effectiveness of BUN as a prognostic marker was further validated using ROC curves for the external validation set (*n* = 49). The AUC of BUN was 0.803 (95% CI, 0.655–0.950; *P* = 0.011). It showed a good ability to predict a 30-day all-cause mortality in patients with SAP. We also observed similar results regarding disease severity, including the Acute Physiology and Chronic Health Evaluation II score (before PSM: 16 [IQR, 8] vs. 8 [IQR, 6], *P* < 0.001; after PSM: 18 [IQR, 10] vs. 12 [IQR, 7], *P* < 0.001), SOFA score (before PSM: 7 [IQR, 5] vs. 3 [IQR, 3], *P* < 0.001; after PSM: 8 [IQR, 5] vs. 5 [IQR, 3.5], *P* < 0.001), and mMarshall score (before PSM: 4 [IQR, 3] vs. 3 [IQR, 1], *P* < 0.001; after PSM: 5 [IQR, 2.5] vs. 3 [IQR, 1], *P* < 0.001). There was significant increase in intensive care unit occupancy in the high BUN level group before PSM (93.3% vs. 73.1%, *P* < 0.001), but not after PSM (97.8% vs. 86.2%, *P* = 0.074).

**Conclusions:**

Our results showed that BUN levels within 24 h after hospital admission were independent risk factors for a 30-day all-cause death in patients with SAP.

## 1. Introduction

Acute pancreatitis (AP) is a digestive system disease with an increased prevalence. The pathogenesis of AP involves the self-digestion of exocrine pancreas due to various reasons. AP is generally a self-limiting illness whose symptoms resolve within several days; however, approximately 20% of cases can progress to severe disease with multiple organ failure and even death. Severe AP (SAP) involves persistent multiple organ failure (POF) in one or more organs with a mortality rate of 15%-30% [1].

Reliable prognostic factors can significantly affect treatment and clinical care. Most previous studies focused on exploring early predicators of progression from AP to SAP or mortality from AP [2–4]. Thus, an easy-to-use predictor of SAP outcomes and severity remains rarely reported.

Here, we aimed to identify a meaningful independent predictor of poor outcomes to improve the prognosis of patients with AP or SAP.

## 2. Method

### 2.1. Study Participants

We reviewed 1608 consecutive cases of AP between April 2017 and May 2021 at Xiangya Hospital, Central South University. Data from 249 patients who diagnosed with SAP according to the modified Atlanta criteria 2012 were used as the training cohort.

From June 2021 to February 2022, all patients with SAP were defined as external patients (*n* = 49) for validation. Those who met at least two of the following criteria were diagnosed with AP: [1] an acute, sudden surge in pain, radiating to the back and waist; [2] high concentration of serum amylase and/or a lipase level ≥ 3 times the normal reference values; and [3] a typical imaging change on abdominal ultrasound, enhanced computed tomography, or magnetic resonance imaging (pancreatic edema or pancreatic exudate). AP with POF > 48 h and a modified Marshall score of ≥2 points was diagnosed as SAP. All patients were divided into a survival group (*n* = 198) and a mortality group (*n* = 51) based on their survival status at a 30-day posthospital admission. A telephone follow-up was conducted to determine a 30-day survival if the patient's hospital stay was <30 days.

### 2.2. Clinical Data Collection

We gathered the following variables for this retrospective analysis: basic information including age, sex, history of smoking and alcohol consumption, comorbidities (diabetes mellitus and hypertension), body temperature, systolic and diastolic blood pressure, heart rate, and respiratory rate. Laboratory tests include arterial blood gases (pH value, FiO_2_, PaO_2_, PaCO_2_, and lactic acid), routine blood examinations (hematocrit; white blood cell, platelet, neutrophil, lymphocyte, eosinophil, basophil, and monocyte counts; mean corpuscular volume; mean corpuscular hemoglobin; mean corpuscular hemoglobin concentration; red blood cell distribution width; plateletcrit; mean platelet volume; and platelet distribution width), routine serum biochemistry (potassium, sodium, and calcium concentrations; albumin, total bilirubin, alanine aminotransferase, aspartate aminotransferase, blood urea nitrogen, creatinine, triglyceride, total cholesterol, high-density lipoprotein cholesterol, and low-density cholesterol levels), blood coagulation tests (plasma prothrombin time, activated partial thromboplastin time, thrombin time, and fibrinogen level), blood and urinary amylase levels, and procalcitonin level. The baseline clinical data were collected within 24 h of admission and analyzed. Other outcome indexes included intensive care unit (ICU) admission rate, incidence of invasive mechanical ventilation (IMV), and length of hospital stay (LOS). Indicators related to disease severity included Acute Physiology and Chronic Health Evaluation (APACHE) II score, Sequential Organ Failure Assessment (SOFA) score, and modified Marshall (mMarshall) score.

### 2.3. Statistical Analysis

Continuous variables are expressed as mean (SD) for normally distributed variables using an unpaired *t*-test and median (IQR) for nonnormal distributions and were compared between groups using the Mann-Whitney *U* test. Categorical variables are presented as count (proportion) and were compared using the chi-square test or Fisher's exact test. First, a univariate logistic analysis was performed to identify latent predictors. Variables with values of *P* > 0.10 were included in the multivariable logistic regression model analysis. Statistical significance was set at *P* < 0.05. The receiver operating characteristic (ROC) curve was used to determine the area under the curve (AUC), sensitivity, and specificity of variables screened by multivariate logistic regression analysis. Propensity score matching (PSM) was used to control for confounding factors between the survival and mortality groups at a ratio of 1 : 1 using the nearest neighbor PSM algorithm. The statistical analysis was performed using SPSS software (version 26.0).

## 3. Results

**Figure 1:**
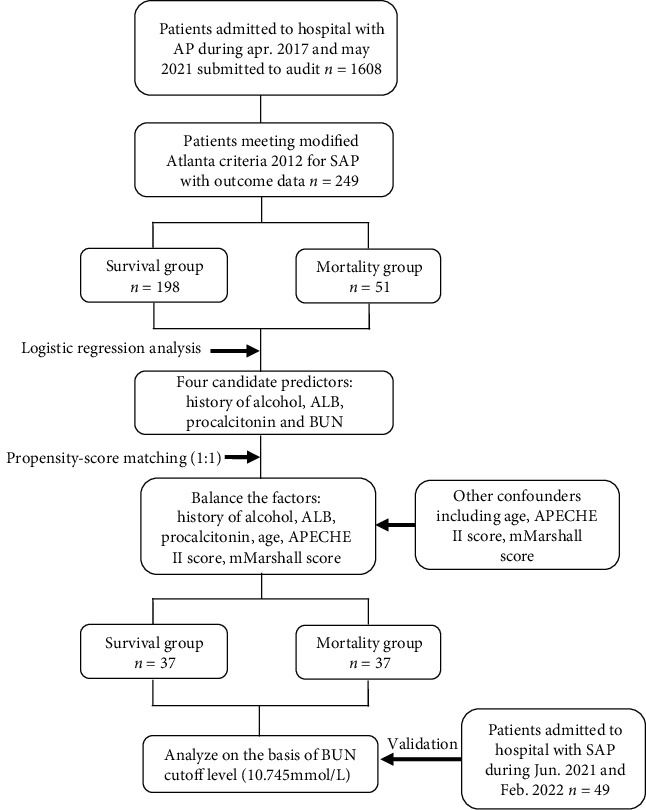
Flow chart of the study.

A total of 1608 patients with AP were admitted to our institution between April 2017 and May 2021; of them, 249 patients with SAP were enrolled (Figure 1(a)). The all-cause mortality rate at a 30-day postadmission was 25.8% (51/198). The baseline characteristics of the 249 SAP patients are presented in Tables 1 and 2. Patients in the mortality group (53.08 ± 13.54 years) were older than those in the survival group (47.24 ± 12.7 years, *P* = 0.007). Statistically significant intergroup differences were noted in 30 parameters, such as BUN (6.685 [IQR, 6.3] mmol/L vs. 20.45 [IQR, 19.7] mmol/L; *P* < 0.001). See Table 3 for tract abbreviations and associated acronyms in Table 1.

Thirty variables with *P* < 0.05 on PSM (Table 1) were subjected to univariate logistic regression analysis (Table 4). Following the exclusion of collinearity, parameters with values of *P* < 0.10 including history of alcohol consumption and BUN, procalcitonin, ALB, and AST levels were entered into the multivariate analysis. Ultimately, four variables were included in the final model. Of them, BUN (odds ratio [OR], 1.097; 95% confidence interval [CI], 1.052–1.144; *P* < 0.001) and procalcitonin (OR, 1.070; 95% CI, 1.035–1.105; *P* < 0.001) levels were risk factors, whereas a history of alcohol consumption (OR, 0.192; 95% CI, 0.064–0.574; *P* = 0.003) and ALB level (OR, 0.829; 95% CI, 0.743–0.92; *P* = 0.001) were protective factors.

**Figure 2:**
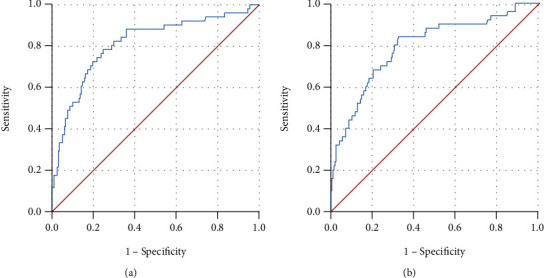
Predictive values of (a) BUN level (AUC: 0.820; 95% confidence interval [CI]: 0.721–0.870; *P* < 0.001) and (b) procalcitonin (AUC: 0.795; 95% [CI]: 0.750–0.890; *P* < 0.001) for a 30-day all-cause mortality.

We further analyzed the ROC curve to determine the diagnostic value of the risk factors including BUN and procalcitonin levels using multivariate logistic regression analysis (Figure 2). The area under the ROC curve for BUN was 0.820 (95% CI, 0.721–0.870; *P* < 0.001; Figure 2(a)). The AUC for procalcitonin was 0.795 (95% CI, 0.750–0.890; *P* < 0.001; Figure 2(b)). The best cut-off values for BUN and procalcitonin were 10.745 mmol/L (sensitivity = 0.780, specificity = 0.751) and 3.0705 ng/mL (sensitivity = 0.837, specificity = 0.678), respectively. In contrast, the potential value of BUN was higher than that of procalcitonin as a predictor of a 30-day all-cause mortality in patients with SAP. Therefore, we used BUN for further validation.

To further verify this conclusion, we matched the predictors in the multivariate logistic regression model except BUN, and continuous variables including ALB and procalcitonin were stratified by the median cut-off point. In addition, confounders that reportedly affect outcomes for AP or SAP, including age, APACHE II score, and mMarshall score, were matched [5–11] (Table 5). After PSM, BUN levels were still higher in the mortality group than in the survival group (18.415 [IQR, 19.555] mmol/L vs. 10.63 [IQR, 6.03] mmol/L; *P* = 0.005). After PSM, there were no statistically significant intergroup differences in the history of alcohol consumption, ALB level, and procalcitonin level (Table 1). In the multivariate logistic regression model, BUN level was the only independent risk factor associated with a 30-day all-cause mortality (Table 4). In addition, the effectiveness of BUN as a prognostic marker was further validated using ROC curves for the external validation set, whose underlying conditions are listed in Table S1. As shown in Figure S1, the AUC of BUN was 0.803 (95% CI, 0.655–0.950; *P* = 0.011). The best cut-off value was 12.01 mmol/L (sensitivity = 0.714, specificity = 0.810). These results showed a good ability to predict a 30-day all-cause mortality in patients with SAP.

Moreover, patients with SAP were grouped by BUN level and stratified by the optimal cut-off value (10.745 mmol/L) based on ROC analysis. We compared the indices related to outcome or disease severity [12–16] between high-level group (BUN > 10.745 mmol/L) and low-level group (BUN ≤ 10.745 mmol/L) (Table 2).

Patients with high BUN levels were associated with significantly higher IMV rate (before PSM: 61.8% vs. 20.6%, *P* < 0.001; after PSM: 71.1% vs. 32%, *P* = 0.048) and 30-day all-cause mortality rates (before PSM: 44.9% vs. 6.9%, *P* < 0.001; after PSM: 60% vs. 34.5%, *P* = 0.032) than those with low BUN levels before or after PSM. We also observed similar results in the indicators of disease severity, including APACHE II score (before PSM: 16 [IQR, 8] vs. 8 [IQR, 6], *P* < 0.001; after PSM: 18 [IQR, 10] vs. 12 [IQR, 7], *P* < 0.001), SOFA score (before PSM: 7 [IQR, 5] vs. 3 [IQR, 3], *P* < 0.001; after PSM: 8 [IQR, 5] vs. 5 [IQR, 3.5], *P* < 0.001) and mMarshall score (before PSM: 4 [IQR, 3] vs. 3 [IQR, 1], *P* < 0.001; after PSM: 5[IQR, 2.5] vs. 3[IQR, 1], *P* < 0.001). There was a significant increase of ICU occupancy in the high BUN level group before PSM (93.3% vs. 73.1%, *P* < 0.001) but not after PSM (97.8% vs. 86.2%, *P* = 0.074). The difference in LOS was not statistically significant regardless of whether PSM was performed.

## 4. Discussion

Due to the high mortality rate among patients with SAP, it is necessary to rapidly identify those with a more severe disease state and a high risk of death early during hospitalization. Our study showed that a high BUN level is a reliable predictor of early warning of SAP. BUN level at admission was the only parameter with an AUC > 0.80 among single predictors for predicting a 30-day all-cause mortality. It was also an independent prognostic factor for SAP before and after PSM. In addition, a high BUN level (>10.745 mmol/L) was associated with an increased risk of IMV and ICU admission.

BUN is produced by the liver and excreted by the kidneys. Previous studies of BUN have primarily focused on renal diseases. More recently, several studies reported that BUN concentrations and their changes can predict AP severity and mortality with high sensitivity [17–20]. However, limited data of SAP outcomes and severities have been reported.

With the exception of pancreatic disease, BUN levels have predictive power as independent or integrated biomarkers such as the incidence of acute cardiac and cerebral vascular events, mortality of critically ill patients, and coronavirus disease 2019 patients [21–25]. Little is known about the predictive ability of BUN beyond the estimation of renal function. There are a few possible explanations for this: [1] renal hypovolemia due to increased vascular permeability and interstitial extravasations induced by inflammation correlated with the systemic inflammatory response [26–28] and [2] chemical injury to the kidney by activated enzymes, inflammatory factors, and cytokines from the blood circulation [29–31].

Several studies have reported on the bedside index for severity in AP (BISAP), a potential prognostic scoring system for identifying patients at high risk for in-hospital mortality [11, 32, 33]. It had a comparable ability to that of APACHE II to predict disease severity, organ failure, and death among patients with AP [33]. We can only exclude it from the disease severity index because its calculation includes BUN levels. In our study, the APACHE II AUC was 0.875 (95% CI 0.831-0.920; *P* < 0.001) (data not shown), higher than that of other variables. However, this method is too complex to be widely used.

Patients with SAP often have comorbid damage to other distant organs. We found that the lung was the most frequently involved organ, affecting accounting for 88.3% of patients with SAP (data not shown). When grouped according to the optimal BUN cut-off value, the incidence of respiratory distress syndrome (ARDS) and acute respiratory failure (ARF) (data not shown) differed no significantly. These results suggest that BUN levels may be independent of severity of AP-induced lung injury severity. Therefore, further studies are required.

## 5. Conclusions

Our results showed that BUN level within 24 h after hospital admission was an independent risk factor for a 30-day all-cause mortality in patients with SAP.

## Figures and Tables

**Table 1:** Basic clinical characteristics before and after propensity score matching.

Variables	Before propensity score matching			After propensity score matching		
	Survival (*n* = 198)	Mortality (*n* = 51)	*P* value	Survival (*n* = 37)	Mortality (*n* = 37)	*P* value
Age, yr, mean (SD)	47.24 (12.70)	53.08 (13.54)	0.007	51.59 (14.893)	52.35 (13.438)	0.819
Gender, male, *n*(%)	137 (62.7)	32 (69.2)	0.379	27 (73)	23 (62.2)	0.321
Smoking, yes, *n*(%)	79 (39.9)	9 (17.6)	0.003	12 (32.4)	8 (21.6)	0.295
Alcohol, yes, *n*(%)	69 (34.8)	9 (17.6)	0.020	7 (18.9)	8 (21.6)	1.000
Hypertension, yes, *n*(%)	53 (26.8)	16 (31.4)	0.512	10 (27)	11 (29.7)	0.797
DM, yes, *n*(%)	53 (26.8)	10 (19.6)	0.367	12 (32.4)	5 (13.5)	0.053
Causes, *n*(%)			0.139			0.832
Hypertriglyceridemia	101 (51.0)	18 (35.3)		15 (40.5)	14 (37.8)	
Gallstone	61 (30.8)	21 (41.2)		12 (32.4)	15 (40.5)	
Others	36 (18.2)	12 (23.5)		10 (27)	8 (21.6)	
FHR, cpm, mean (SD)	110.22 (21.63)	112.18 (18.98)	0.555	114 (28)	112 (25)	0.733
TEMP, °C, median (IQR)	37.00 (1.10)	37.3 (1.3)	0.124	37 (0.75)	37.2 (1.35)	0.212
RR, cpm, median (IQR)	23.00 (8.00)	23 (11)	0.947	25 (11)	23 (11)	0.672
SBP, mmHg, mean (SD)	137.39 (23.66)	124.39 (28.13)	0.001	141.03 (25.389)	125.49 (27.602)	0.014
DBP, mmHg, median (IQR)	80.00 (17.25)	74 (21)	0.001	88 (23)	74 (19.1)	0.004
FiO_2_, median (IQR)	0.4 (0.17)	0.41 (0.2)	0.000	0.4 (0.27)	0.4 (0.2)	0.287
PaO_2_, KPa, median (IQR)	74 (23.25)	81 (62)	0.006	77 (26)	78 (42)	0.563
PaCO_2_, KPa, median (IQR)	35 (8)	35 (8)	0.993	35 (9.45)	35 (7)	0.611
Lac, mmol/L, median (IQR)	1.4 (0.9)	1.85 (1.0)	0.000	1.3 (0.6)	1.8 (1.3)	0.001
WBC, ×10^9^/L, median (IQR)	12.4 (8.2)	13.2 (8.1)	0.597	12.4 (10.15)	13.1 (8.75)	0.733
HCT, %, mean (SD)	33.62 (8.25)	28.19 (8.90)	0.000	31.2 (10)	27.5 (11.1)	0.109
PLT, G/L, median (IQR)	185 (114.25)	158 (131)	0.044	200 (111)	158 (122)	0.058
NEc, G/L, median (IQR)	10.5 (8)	11.2 (9.5)	0.647	10.3 (10.6)	10.2 (8.6)	0.661
LYc, G/L, median (IQR)	0.8 (0.5)	0.7 (0.5)	0.016	0.8 (0.55)	0.8 (0.65)	0.248
EOc, G/L, median (IQR)	0 (0.1)	0 (0.1)	0.513	0 (0.1)	0 (0.1)	0.668
BAc, G/L, median (IQR)	0 (0.02)	0 (0.01)	0.693	0 (0.02)	0 (0.03)	0.501
MOc, G/L, median (IQR)	0.6 (0.6)	0.6 (0.8)	0.749	0.7 (0.5)	0.6 (0.8)	0.692
MCV, fl, median (IQR)	91.7 (7)	92.6 (7)	0.828	91.3 (7.4)	92.7 (6.55)	0.310
MCH, pg, median (IQR)	30.5 (2.5)	30.7 (2.2)	0.883	30.5 (3.1)	30.7 (1.4)	0.414
MCHC, g/L, median (IQR)	331 (15)	331 (18.1)	0.761	330 (16.7)	332 (17.91)	0.523
PCT, %, median (IQR)	0.18 (0.1)	0.17 (0.12)	0.134	0.18 (0.1)	0.17 (0.12)	0.178
RDW, %, median (IQR)	14.2 (1.6)	15.3 (1.5)	0.000	14.5 (1.6)	15.2 (1.8)	0.030
MPV, fl, median (IQR)	9.51 (1.8)	9.9 (2.08)	0.053	9.5 (2)	10.1 (1.93)	0.119
K, mmol/L, median (IQR)	3.715 (0.7)	4.05 (0.8)	0.000	3.96 (0.77)	4.01 (0.72)	0.191
Na, mmol/L, median (IQR)	140.25 (6.5)	144.1 (7.9)	0.000	140.9 (8.45)	144.2 (7.65)	0.021
Ca, mmol/L, median (IQR)	1.94 (0.3)	1.96 (0.3)	0.466	1.94 (0.37)	1.96 (0.37)	0.489
ALB, g/L, median (IQR)	30.3 (6.1)	26.5 (5.1)	0.000	28.5 (6.7)	27.9 (5.6)	0.077
TBIL, *μ*mol/L, median (IQR)	19.4 (22)	25.4 (65.5)	0.025	19.4 (19.3)	25.4 (57.4)	0.355
ALT, U/L, median (IQR)	23.5 (29.2)	27.1 (50.6)	0.517	27.9 (34.45)	32.6 (72.3)	0.292
AST, U/L, median (IQR)	36.1 (33.7)	55 (82.6)	0.009	45.1 (43.75)	56.2 (103.25)	0.196
BUN, mmol/L, median (IQR)	6.685 (6.3)	20.45 (19.7)	0.000	10.14 (11.25)	18.14 (19.24)	0.007
CREA, *μ*mol/L, median (IQR)	76.55 (53.6)	298.7 (272.1)	0.000	102.9 (198.6)	297.1 (309.6)	0.008
TG, mmol/L, median (IQR)	2.98 (3.75)	2.5 (3.17)	0.700	2.75 (4.02)	2.5 (3.57)	0.829
TC, mmol/L, median (IQR)	3.69 (2.83)	2.45 (1.74)	0.000	2.76 (2.46)	2.5 (2.16)	0.177
HDL-C, mmol/L, median (IQR)	0.62 (0.46)	0.37 (0.29)	0.000	0.58 (0.39)	0.41 (0.28)	0.005
LDL-C, mmol/L, median (IQR)	2.37 (1.74)	1.48 (1.05)	0.000	2.01 (1.63)	1.59 (1.39)	0.079
APTT, s, median (IQR)	33.8 (9.1)	37.2 (9.3)	0.007	35.2 (9.38)	37.2 (12.4)	0.289
PT, s, median (IQR)	13.9 (2.55)	14.8 (2.8)	0.041	14.25 (2.68)	14.8 (2.95)	0.222
TT, s, median (IQR)	16.66 (3.58)	16 (3.5)	0.479	16.22 (4.08)	15.8 (3.25)	0.449
FIB, g/L, median (IQR)	5.76 (2.92)	4.38 (2.21)	0.000	5.93 (2.97)	3.99 (1.95)	0.004
DD, *μ*g/mL, median (IQR)	2.49 (3.89)	4.14 (8.49)	0.007	3.48 (8.02)	4.14 (10.32)	0.149
Procalcitonin, ng/mL, median (IQR)	1.65 (4.25)	10.83 (30.34)	0.000	5.63 (12.02)	8.32 (33.225)	0.189

^∗^ *P* < 0.05 and ^∗∗^*P* < 0.001. Definitions of abbreviations are shown in Table 3.

**Table 2:** Outcomes and severity of patients with SAP based on BUN level.

Variables	Before propensity score matching			After propensity score matching		
	BUN ≤ 10.745 mmol/L (*n* = 160)	BUN > 10.745 mmol/L (*n* = 89)	*P* value	BUN ≤ 10.745 mmol/L (*n* = 29)	BUN > 10.745 mmol/L (*n* = 45)	*P* value
mMarshall score, median (IQR)	3 (1)	4 (3)	0.000	3 (1)	5 (2.5)	0.000
APACHE II score, median (IQR)	8 (6)	16 (8)	0.000	12 (7)	18 (10)	0.000
SOFA score, median (IQR)	3 (3)	7 (5)	0.000	5 (3.5)	8 (5)	0.000
SIRS score, median (IQR)	6 (5)	7 (5)	0.000	6 (4.5)	7 (4)	0.011
30-day all-cause mortality, *n*(%)	11 (6.9)	40 (44.9)	0.000	10 (34.5)	27 (60)	0.032
ICU admission rate, *n*(%)	117 (73.1)	83 (93.3)	0.000	25 (86.2)	44 (97.8)	0.074
LOS, *d*, median (IQR)	18 (18)	18 (27)	0.505	22 (26)	17 (26)	0.184
IMV, *n*(%)	33 (20.6)	55 (61.8)	0.000	14 (32)	32 (71.1)	0.048

mMarshall score: modified Marshall score; APACHE II score: Acute Physiology and Chronic Health Evaluation II score; SOFA: Sequential Organ Failure Assessment; SIRS: systemic inflammatory response syndrome; ICU: intensive care unit; LOS: length of stay; IMV: invasive mechanical ventilation.

**Table 3:** Definition of abbreviations.

Sequence	Abbreviation	Meaning
1	ALB	Albumin
2	ALT	Alanine aminotransferase
3	APTT	Activated partial thromboplastin time
4	AST	Aspartate aminotransferase
5	Bac	Basophils
7	BUN	Blood urea nitrogen
8	Ca	Calcium concentration
9	CREA	Creatinine
10	DBP	Diastolic blood pressure
11	DD	D-dimer
12	DM	Diabetes mellitus
13	Eoc	Eosinophils
14	FDP	Fibrin degradation product
15	FHR	Heart rate
16	FIB	Fibrinogen
17	FiO_2_	Fraction of inspiration oxygen
18	GLU	Glucose
19	HCT	Hematocrit
20	HDL-C	High-density lipoprotein cholesterol
21	INR	International normalized ratio
22	K	Potassium concentration
23	Lac	Lactic acid
24	LDH	Lactate dehydrogenase
25	LDL-C	Low-density cholesterol
26	LYc	Lymphocytes
27	MCH	Mean corpuscular hemoglobin
28	MCHC	Mean corpuscular hemoglobin concentration
29	MCV	Mean corpuscular volume
30	Moc	Monocytes
31	MPV	Mean platelet volume
32	Na	Sodium concentration
33	NEc	Neutrophils
34	PaCO_2_	Artery carbon dioxide partial pressure
35	PaO_2_	Arterial oxygen partial pressure
36	PCT	Plateletcrit
37	PDW	Platelet distribution width
38	PLT	Platelets
39	PT	Plasma prothrombin time
40	RBC	Red blood cells
41	RDW	Red blood cell distribution width
42	RR	Respiratory rate
43	SBP	Systolic blood pressure
44	TBIL	Total bilirubin
45	TC	Total cholesterol
46	TEMP	Temperature
47	TG	Triglyceride
48	TT	Thrombin time
50	WBC	White blood cell

**Table 4:** Predictors for mortality of patients with SAP in logistic regression analysis.

Variable	Univariate analysis				Multivariable analysis				After propensity score matching			
	OR	Lower	Upper	*P* value	OR	Lower	Upper	*P* value	OR	Lower	Upper	*P* value
Alcohol	0.137	0.027	0.696	0.017	0.192	0.064	0.574	0.003				
ALB	0.843	0.730	0.973	0.020	0.829	0.743	0.925	0.001				
BUN	1.067	0.988	1.153	0.098	1.097	1.052	1.144	0.000	1.070	1.017	1.126	0.009
Procalcitonin	1.054	1.009	1.101	0.018	1.070	1.035	1.105	0.000				
HDLC	0.079	0.005	1.282	0.074								
AST	1.003	0.999	1.007	0.131								
Smoking	3.317	0.073	150.255	0.538								
Age	1.020	0.975	1.066	0.394								
SBP	1.000	0.974	1.027	0.984								
DBP	0.998	0.957	1.040	0.917								
FiO_2_	0.555	0.024	12.790	0.713								
PaO_2_	1.006	0.988	1.024	0.513								
Lac	1.214	0.715	2.062	0.473								
PLT	1.002	0.998	1.005	0.329								
HCT	0.991	0.916	1.073	0.825								
LYc	0.442	0.156	1.251	0.124								
RDW	0.930	0.722	1.200	0.578								
K	2.388	0.835	6.829	0.104								
Na	1.062	0.961	1.173	0.236								
TBIL	1.004	0.999	1.010	0.136								
CREA	1.001	0.996	1.005	0.750								
TC	1.085	0.496	2.371	0.839								
LDLC	1.086	0.269	4.378	0.907								
PT	0.987	0.753	1.292	0.922								
APTT	0.978	0.935	1.022	0.326								
FIB	0.901	0.712	1.139	0.383								
DD	0.976	0.882	1.079	0.631								

Definitions of abbreviations are shown in Table 3.

**Table 5:** Balance the factors for propensity score matching.

Variable	Before propensity score matching			After propensity score matching		*P* value
	Survival (*n* = 198)	Mortality (*n* = 51)	*P* value	Survival (*n* = 37)	Mortality (*n* = 37)	
Age, yr, *n*(%)			0.023			0.642
≤50	124 (62.6)	23 (45.1)		17 (45.9)	19 (51.4)	
>50	74 (37.4)	28 (54.9)		20 (54.1)	18 (48.6)	
Alcohol, yes, *n*(%)	69 (34.8)	9 (17.6)	0.020	7 (18.9)	8 (21.6)	1.000
APACHE II, *n*(%)			0.000			1.000
≤8	86 (43.4)	3 (5.9)		3 (8.1)	3 (8.1)	
>8	112 (56.6)	48 (94.1)		34 (91.9)	34 (91.9)	
mMarshall score, *n*(%)			0.000			0.787
≤3	153 (77.3)	19 (37.3)		10 (27.0)	8 (21.6)	
>3	45 (22.7)	32 (62.7)		27 (73.0)	29 (78.4)	
ALB, g/L, *n*(%)			0.000			0.809
≤29.4	87 (43.9)	38 (74.5)		23 (62.2)	24 (64.9)	
>29.4	111 (56.1)	13 (25.5)		14 (37.8)	13 (35.1)	
Procalcitonin, ng/mL, *n*(%)			0.000			1.000
≤2.22	116 (58.6)	9 (7.2)		10 (27.0)	9 (24.3)	
>2.22	82 (41.4)	42 (82.4)		27 (73.0)	28 (75.7)	

mMarshall score: modified Marshall score; APACHE II score: Acute Physiology and Chronic Health Evaluation II score; ALB: albumin.

## Data Availability

The data for this study are available from the authors upon reasonable request with permission from Xiangya Hospital, Central South University.

## Supplementary Materials

Supplementary Materials Table S1: baseline characteristics of the training cohort (*n* = 249) and validation cohort (*n* = 49), including age, gender, alcohol history, ALB, procalcitonin, BUN levels, mMarshall score, APACHE II score, and an all-cause 30-day mortality. There were no significant differences between these two cohorts except ALB level and APACHE II score. Figure S1: the receiver operating characteristic (ROC) curve of BUN level in the validation cohort (AUC: 0.803; 95% confidence interval [CI]: 0.655–0.950; *P* = 0.011). The best cut-off value was 12.01 mmol/L (sensitivity = 0.714, specificity = 0.810).
