# Supplementary material for: Blood Urea Nitrogen as a Prognostic Marker in Severe Acute Pancreatitis
Source: Dis Markers. 2022 Mar 29;2022:7785497. doi: 10.1155/2022/7785497 (PMC8983180; doi:10.1155/2022/7785497)
Supplement: Supplementary Materials — Table S1: baseline characteristics of the training cohort (n = 249) and validation cohort (n = 49), including age, gender, alcohol history, ALB, procalcitonin, BUN levels, mMarshall score, APACHE II score, and an all-cause 30-day mortality. There were no significant differences between these two cohorts except ALB level and APACHE II score. Figure S1: the receiver operating characteristic (ROC) curve of BUN level in the validation cohort (AUC: 0.803; 95% confidence interval [CI]: 0.655–0.950; P = 0.011). The best cut-off value was 12.01 mmol/L (sensitivity = 0.714, specificity = 0.810). [file 7785497.f1.docx]

Table S1. Baseline characteristics of the training cohort and validation cohort.

| Variables | Training cohort (n=249) |  | Validation cohort (n=49) | P-value |
| --- | --- | --- | --- | --- |
| Age, yr, median (IQR) | 48(16) |  | 45(21) | 0.154 |
| Gender, male, n(%) | 169(67.9) |  | 33(67.3) | 0.943 |
| Alcohol, yes, n(%) | 78(31.3) |  | 17(34.7) | 0.644 |
| ALB, g/L, median (IQR) | 29.4(6.35) |  | 31.9(8.45) | 0.048 |
| Procalcitonin, ng/ml, median (IQR) | 2.32(8.23) |  | 1.28(6.103) | 0.068 |
| BUN, mmol/L, median (IQR) | 7.66(10.55) |  | 5.88(7.885) | 0.146 |
| mMarshall score, median (IQR) | 3(2) |  | 3(2) | 0.865 |
| Apache II score, median (IQR) | 11(9) |  | 9(9) | 0.039 |
| All-cause day-30 mortality, n(%) | 51(20.5) |  | 7(14.3) | 0.32 |


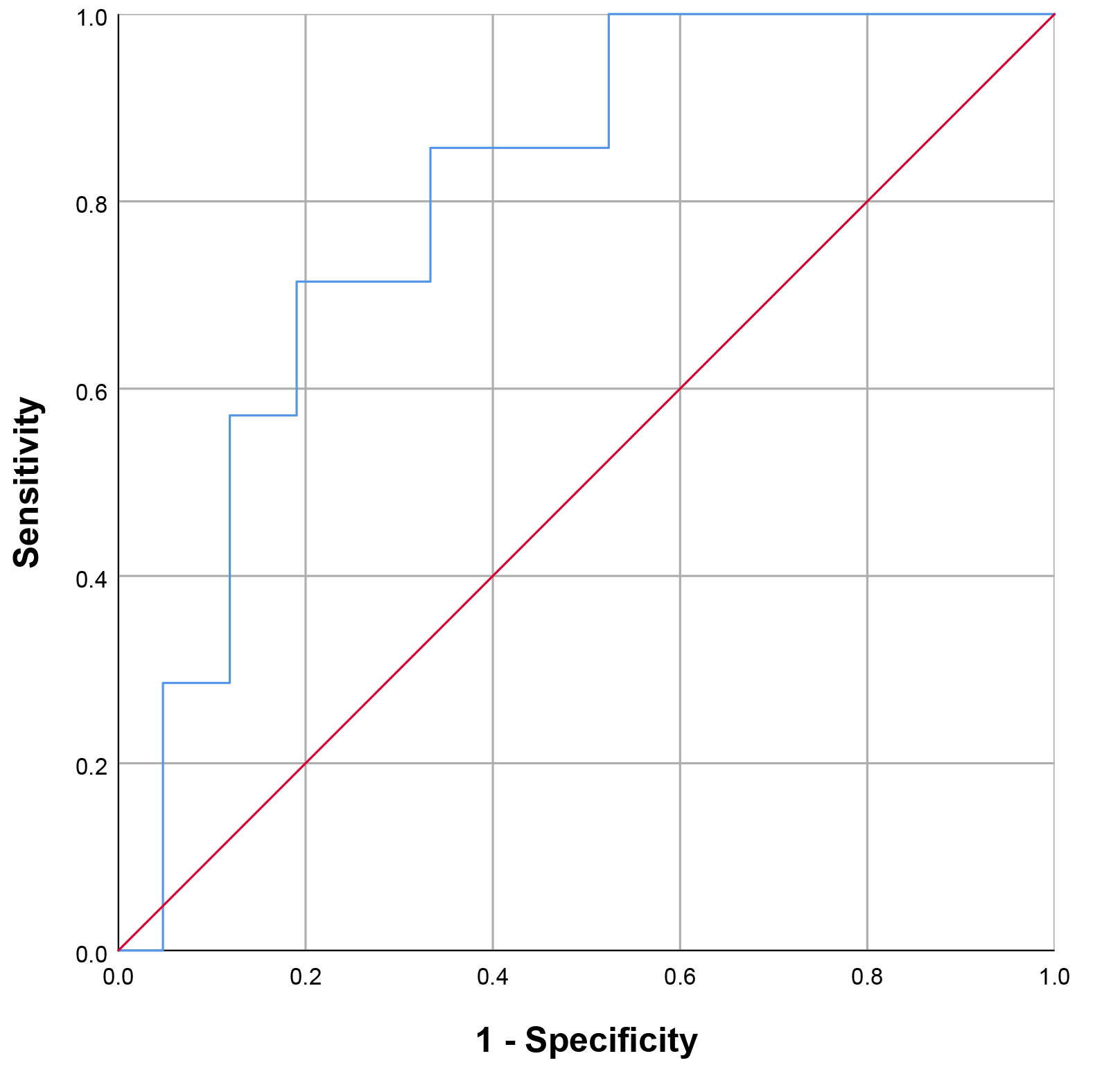


Figure S1. Predictive values of BUN level in the validation cohort (AUC: 0.803; 95% confidence interval [CI]: 0.655–0.950; p=0.011)
